# Remediating lapses in professionalism among undergraduate pre-clinical medical students in an Asian Institution: a multimodal approach

**DOI:** 10.1186/s12909-018-1206-2

**Published:** 2018-05-02

**Authors:** Ardi Findyartini, Nani Cahyani Sudarsono

**Affiliations:** 1 Department of Medical Education, Faculty of Medicine Universitas Indonesia, Jakarta, Indonesia; 2 Department of Community Medicine, Faculty of Medicine Universitas Indonesia, Jakarta, Indonesia

**Keywords:** Professionalism, Remediation, Undergraduate, Instructional design, Medicine

## Abstract

**Background:**

Fostering personal identity formation and professional development among undergraduate medical students is challenging. Based on situated learning, experiential learning and role-modelling frameworks, a six-week course was developed to remediate lapses in professionalism among undergraduate medical students. This study aims to explore the students’ perceptions of their personal identity formation and professional development following completion of the course.

**Methods:**

This qualitative study, adopting a phenomenological design, uses the participants’ reflective diaries as primary data sources. In the pilot course, field work, role-model shadowing and discussions with resource personnel were conducted. A total of 14 students were asked to provide written self-reflections. Consistent, multi-source feedback was provided throughout the course. A thematic analysis was conducted to identify the key processes of personal and professional development among the students during remediation.

**Results:**

Three main themes were revealed. First, students highlighted the strength of small group activities in helping them ‘internalise the essential concepts’. Second, the role-model shadowing supported their understanding of ‘what kind of medical doctors they would become’. Third, the field work allowed them to identify ‘what the “noble values” are and how to implement them in daily practice’.

**Conclusion:**

By implementing multimodal activities, the course has high potential in supporting personal identity formation and professional development among undergraduate pre-clinical medical students, as well as remediating their lapses in professionalism. However, there are challenges in implementing the model among a larger student population and in documenting the long-term impact of the course.

## Background

Medicine is a noble profession, aiming to serve communities and requires ‘mindful practice’ [1, 2]. Medical education programmes must therefore assure that their graduates are not only competent in implementing relevant knowledge and can perform the correct clinical procedures for each health problem (i.e. ‘doing the right thing’), but that they are also prepared to approach each health problem from multiple perspectives, including communication, teamwork, leadership and information technology (i.e. ‘doing the thing right’). Last, but not least, future graduates must be prepared to practise medicine professionally (i.e. ‘be the right person doing it’) [3].

There are several definitions for professionalism. According to a systematic review by Wilkinson et al., professionalism is comprised of five key aspects: reliability; adherence to ethical principles; effective interactions with patients, their family members and their significant others; effective interactions within the healthcare system; and a commitment to improving the competence of oneself, others and the healthcare system [4]. Irby et al. argued that professionalism should be considered as a set of ethical values and attributes (virtue-based professionalism), a set of behaviours (behaviour-based professionalism) or a method for personal identity formation; these three views are based on different constructs that can deepen one’s understanding of professionalism [5]. In addition to these definitions, medical professionalism is a dynamic construct that requires practitioners to picture themselves providing excellent, ethical and altruistic patient care and satisfying the expectations of those outside the profession [1].

Due to challenges in defining professionalism, medical education programmes have no other option than to teach it explicitly [1, 5]. This is strongly supported by studies highlighting that students with identified unprofessional behaviours may be at risk of disciplinary action when they become practising physicians [6, 7]. Various curriculum designs and teaching/learning methods have been suggested to foster the personal and professional development of medical students [5]. Such efforts should aim at both preventing students from developing unprofessional behaviours as well as remediating lapses in professionalism [8]. Unprofessional behaviours among students range from mild to serious [8, 9], including lapses in responsibility, lapses in their relationships with other healthcare practitioners and lapses in their relationships with patients [9]. Ziring et al. also stated that regardless of the type of lapse, it is important to realise that medical students are not yet professionals. Therefore, when lapses in professionalism occur, students need to undergo remediation rather than be punished [9].

Remediation should help students develop a strong understanding of the cognitive component of professionalism, which requires explicit teaching [10]. Special curricula or assignments are among the most common remediation strategies, in addition to mandated mental health evaluation, mandated professionalism mentoring, stress-management counselling, course repetition and community service [9]. In the long term, however, remediation curricula should allow for internalisation and socialisation in authentic contexts through situated learning [11]. The learning process must transform novice and non-expert members of the community (i.e. the medical students) into expert members of the medical profession with the appropriate competencies and a commitment to ethical values [10].

Role modelling by experts contributes to the personal and professional development of medical students, creating opportunities for students to observe professionalism in practice [12]. Regarding personal and professional identity formation as part of professional development, the learning process requires self-reflection so that students may internalise the values of professionalism; learn from their experiences; and take further action to improve their understandings, attitudes and behaviours [13, 14]. Narrative reflection is also suggested as a way to encourage students to make conscious efforts towards understanding the complexity of professionalism [15]. A systematic review conducted by Guraya et al. revealed that even though there is no single best model to integrate the teaching of professionalism into the curriculum, the most powerful strategies to teach medical professionalism are role modelling, mentoring, hidden curriculum, reflective practice and effective communication [16]. This further underlines the process of socialisation in the personal and professional identity development of medical students [17].

Developing professionalism and remediating lapses among pre-clinical undergraduate medical students can be challenging due to the level of understanding required and the context of practice. Teaching and learning professionalism in the pre-clinical stage of undergraduate medical education should situate the requisite ethics, values and beliefs, as well as their impacts to personal and professional development, within students’ daily experiences, which are not always related to clinical practice [18]. In addition, some studies show the importance of considering the characteristics of the millennial generation when developing professionalism as a part of medical education [19–22]. The curriculum and teaching/learning process related to this must also consider the specific contexts surrounding the students, including culture. For example, students and academic staff in different organisational cultures may have different understandings of what is and is not a professional lapse in academic integrity (e.g. completing work for another student, altering or manipulating data, etc.) [23]. Studies have also shown that different cultures may have distinctive definitions for some aspects of professionalism [24–26], which, in turn, may cause the teaching of professionalism to vary widely [4, 27].

To date, few studies have focused on describing personal identity and professional development in Asian medical schools [28–30]. Reports on the best practices for remediating lapses in professionalism among medical students are also rare [31]. Given the dynamic development of professionalism and the need to explore various contextual factors, including generational and cultural characteristics, the present study aims to explore students’ perceptions of their personal identity formation and professional development after completing a remediation course in a medical school in Indonesia. The pilot course included situated learning, experiential learning and role modelling as key frameworks.

## Methods

### Context

Professionalism is one of the competencies taught to Indonesian medical graduates [32]. Every medical school in Indonesia, including the Faculty of Medicine at Universitas Indonesia (FMUI), has implemented various approaches to instilling and developing this competency among its students. At FMUI, a 5.5-year curriculum, comprised of 3.5 years of pre-clinical study and 2 years of clinical study, has been offered since 2012. Some courses in the pre-clinical curriculum are dedicated to the personal identity formation and professional development of medical students, whereas during the clinical studies, the curriculum is more embedded in clinical rotations at teaching hospitals and in community-based health services. Despite these opportunities to develop professionalism, there has been feedback from students and academic staff, as well as reports, on the misconduct of medical students, which encouraged the present authors to reflect on FMUI’s curriculum.

Professionalism is a complex construct to teach [33], requiring the preparedness of not only students but also academic staff and institutions. FMUI has implemented strategies to remediate students with confirmed lapses in professionalism, including counselling, community service, course repetition and even suspension. However, no systematic remediation processes exist in the form of a college course, as piloted in the present study. The remediation course was aimed at facilitating self-reflections among medical students with confirmed lapses in professionalism, thereby allowing them to identify what went wrong and how to remediate their behaviours.

### Description of the remediation course

A six-week remediation course was developed, and it was implemented as a pilot course from August–September 2016. The activities were arranged in small group formats. Students had some sessions comprised of topic discussion and role-play with the resource personnel, as well as role-model shadowing and field work. The topics covered professionalism, noble values for medical students and future medical doctors, and applications in daily life. Students were also asked to write a series of reflective diaries, including: daily reflections, a final reflection at the end of the module, reflections on their role-model shadowing and reports on their field activities. During group discussions, students were also encouraged to share their individual reflections as well as their views on several hypothetical situations concerning professionalism and professional lapses. All of their self-reflections were given feedback, both in written and verbal forms, by the authors of this study and other personnel involved in the course. The course description is provided in Table 1.

**Table 1:** Course description

Week	Topics	Activities	Hours
I	The noble values of the medical profession	Group discussion with triggers	4
	The Hippocratic oath and the values of the medical profession	Group discussion with triggers	2
	The history of medicine and the medical profession	Interactive lecture	2
	Professionalism and professional attributes	Interactive lecture	2
	Indonesia Medical Graduates Competence standards: Noble professionalism and self- reflective capabilities	Group discussion with triggers	2
	Individual learning on related topics		6
	*End of the week: Submission of written diaries/reflections*		
II	Role modelling and its role in professional development	Group discussion with triggers	2
	The noble values of the medical profession and FMUI’s values: Implementation in daily practice	Group discussion with triggers	4
	The noble values of the medical profession and FMUI’s values: Reflections	Group discussion with triggers	2
	Individual learning and reflections		20
	*End of the week: Submission of written diaries/reflections*		
III	The noble values of the medical profession and FMUI’s values: Reflections on student organisation activities	Group discussion with triggers	2
	Development of field-project proposals	Group work (two students in each group)	15
	Field-project proposal	Presentation and feedback	6
	Orientation on role-model shadowing activities	Interactive lecture	1
	*End of the week: Submission of written diaries/reflections*		
IV	Role-model shadowing	(By appointment)	8
	Field work		17
	Progress report	Presentation and feedback	4
	*End of the week: Submission of written diaries/reflections*		
V	Role-model shadowing	(By appointment)	8
	Field work		8
	Feedback	Discussion and roleplay	4
	*End of the week: Submission of written diaries/reflections*		
VI	Field report completion	Group work	12
	Portfolio completion	Individual work	12
	Final report	Presentation and feedback	6
	*End of the week: Submission of written diaries/reflections*		

A total of 22 academics with medical backgrounds were involved in the course as resource personnel or tutors (8), as well as role models who the students shadowed (14). These teachers were selected by the course organiser and have been considered positive role models by the undergraduate medical students. The resource personnel and tutors were comprised of experienced medical teachers who facilitated group discussions on the given topics; triggered discussions linked to professional values and the daily practice of professionalism; and provided feedback on the students’ reading summaries, written assignments and reports, as well as their written reflections. The role models were comprised of clinicians, researchers and/or policy makers. During the course, each role model was shadowed by one student in all clinical, educational or organisational activities for at least eight hours. At the end of each activity, the role models provided feedback to the students and encouraged them to reflect on their experiences and ask relevant questions.

In addition to the small group activities and role-model shadowing, groups of two were formed to propose and implement field projects. The students were given freedom to choose project themes that they thought best highlighted the noble values of the medical profession, such as compassion, care for others, integrity and excellence. The proposed themes included health education programmes for schoolchildren and their teachers, health education and general education programmes for street children and children in orphanages, entertainment for children in outpatient care at FMUI’s teaching hospital, exploration of humanities in medicine and student body leadership programmes, and the development of mobile applications to educate fellow medical students on the professional use of social media. The students proposed and discussed their projects with the resource personnel prior to implementation. Each project had to be completed in at least 25 h, which was determined based on the time availability of the course. Once they completed their projects, each group developed a report that was presented during a plenary session attended by all resource personnel.

### Study design

This qualitative study adopted a phenomenological design [34], using reflective diaries as primary data sources. Phenomenology is a qualitative research approach which aims to explore the common lived experiences of a particular group. In addition to interviews with the informants, this approach may also use observation, documentation and other sources [34]. In the present study, the design allowed the authors to explore the meanings and common features perceived by students and teachers who were involved in the remediation course [35]. During the course, students were asked to write daily reflections, role-modelling reports and group fieldwork reports. Tutors and role models provided written feedback for the students. The written reflections were then analysed by the authors of the present study. During analysis, both authors considered the students’ expressions concerning their lived experiences while they learned and practised multiple aspects of professionalism in the remediation course. Although the written reflections were the main sources of data, the authors also recorded the group discussions and their day-to-day observations of the students’ activities to guide the analysis. Interactions between the authors and the students during the course allowed for further understanding of the students’ written reflections. Feedback from the tutors and role models were used to confirm or deny the results.

### Participants

A total of 14 students in the first to third years of their medical studies were involved in this research. The students were assigned to this course due to some lapses in professionalism they had committed over the past semester, namely lapses in responsibility (i.e. inappropriate use of social media and misconduct in organising student activities) [9]. Given the formal curriculum on professionalism for pre-clinical year students, as previously described, the authors believe that the students who enrolled in the remediation course had a general understanding of the key concepts of professionalism. However, the students were not yet able to transfer their understandings to their daily practices as medical students and to how these practices would affect their future professional development as medical doctors. In addition, despite their different levels of study, the students who participated in the remediation course were considered to be at the same stage of professional identity formation, which is ‘the imperial stage’ [17]. At this stage, individuals can assume a professional role; however, it is not fully integrated into their identity [17].

After receiving the students’ verbal consent, the authors explored their written reflections and reports for the purposes of this study. The use of verbal consent was chosen to avoid potential pressure that might happen should students provide written consents. The present authors expected that students were able to express their experience during the remediation course freely. The students were assured that their data will be kept confidential and all quotations derived from their written reflections or reports will be attributed to a pseudonym, thereby mitigating the possibility for them to be individually recognised. The students were also informed that their participation in the study would not influence any course evaluation processes.

### Data collection and analysis

All written reflections and reports were collected during the six-week course. The authors read all the data to identify the key features of the course that the students considered beneficial for their personal identity formation and professional development. A thematic analysis was completed by the two authors independently, and the results were then compared. Further discussions were conducted to resolve any disagreements. Quotations reflecting the themes and sub-themes were also identified and examined for how frequently they were raised by the students. The overall protocol of the study, including the use of verbal consent from the participants, was approved by the Research Ethics Committee of the Faculty of Medicine, Universitas Indonesia.

## Results

The characteristics of the 14 students who were involved in this study are provided in Table 2.

**Table 2:** Characteristics of the informants

No.	Coded initials	Year of study	Gender
1	AA	3	Male
2	BB	3	Male
3	CC	3	Female
4	DD	2	Female
5	EE	2	Female
6	FF	1	Male
7	GG	1	Male
8	HH	1	Female
9	II	1	Female
10	JJ	1	Male
11	KK	3	Female
12	LL	3	Female
13	MM	3	Female
14	NN	3	Female

### The analysis revealed three major themes

1. Internalisation of essential professional values and personal identity formation;
2. Role-model shadowing and understanding of what kind of professional they should become; and
3. Field work as experiential learning and a means to implement professional values in daily practice.

### Internalisation of essential professional values and personal identity formation

A total of 20 expressions from 11 students highlighted their realisations about themselves as current medical students and future doctors. Generally, the 14 students, despite coming from their first three years of medical education, were aware that humanism, professionalism and other abstract values are very important for them to learn. However, they admitted that it had been quite a struggle for them to do so in other parts of the curriculum. They were not sure whether they understood the concepts correctly. During the remediation course, close interactions with the teachers enabled in-depth discussions which facilitated the students’ internalisation of the abstract values they had been struggling with, as evidenced in the following quotation:

*‘…I was really inspired by what Prof A said during our discussion about how to differentiate between “human” and “person”. The latter is a more suitable way of viewing our patients in the doctor–patient relationship. Humanism can be defined as a fundamental viewpoint intended to give meaning to the role of a person as a whole human being who has thoughts, feelings, a mind and a soul.’* (CC, 3rd-year medical student, field work report).

An emergence of self-awareness has also been highlighted by the students. According to them, the daily reflections, completed individually and in groups, contributed to this realisation. While reflecting, they were encouraged to discover more about who they are, which is an essential stepping stone in personal identity formation. For example:

*‘The course has allowed me to recognise more about myself. I feel changed, since I am more sensitive and reflective of all that is happening around me. I am also better able to analyse the cause and effect of something, thus I am able to make better decisions. Besides this, I have become more aware of my bad coping mechanisms and how to fix them*.’ (EE, 2nd-year medical student, final reflection).

Finally, the students appreciated the small group activities in the course, which very supportive in developing their understanding of and internalising the abstract values related to being a good doctor. Students also thought that the openness and freedom of speech in the course gave them more opportunities to internalise the values. In the hierarchical culture of medical schools, especially in Asian countries, both openness and freedom of speech are not the norm in most cases. Regarding the group activities, one student noted:

*‘The discussions were very conducive and interactive. I took valuable lessons from the course, which were learned not only from the discussions but also from the professional and supportive attitudes of the teachers and my friends. I believe all of these aspects form the core of my positive behavioural changes.’* (JJ, 1st-year medical student, final reflection).

### Role-model shadowing and understanding of what kind of professional they should become

Each student had a role-model shadowing activity, which lasted a minimum of eight hours, with academics nominated by the course organiser. A total of 16 comments were identified from students’ written reflections and reports regarding the significance of role-model shadowing to help students understand ‘what to become’. The students observed the application of good values in the daily lives of medical doctors, clinicians, clinical teachers and academics, and healthcare leaders, as shown in the following quotation:

*‘…To be honest, I did not know how we could understand other people’s feelings without feeling them for ourselves. It was when I shadowed Prof B while he visited his patients in the ICU that I finally understood.’* (HH, 1st-year medical student, role-model shadowing report).

The role models whom the students shadowed were very busy, yet the students found they were very passionate and committed to what they were doing. For example:

*‘So, it is possible to become an academic, a clinician, a researcher and a writer in one complete package. He is proof! This really motivates me.’* (LL, 3rd-year medical student, role-model shadowing report).

The role models also showed excellent communication skills and a sincerity to help others; two attributes found in good medical doctors. Students who had not been sufficiently exposed to real clinical settings during the first three years of their medical studies gained valuable lessons from their role models. Overall, the role-model shadowing activity gave students contextual descriptions for what kind of practitioner they should become in the future. For example:

*‘After I saw how he interacts with people, I think it is very important for a doctor to be able to communicate well with others. With proper communication, a patient can better understand what his/her doctor says. In the end, the patient will trust his/her doctor more.’* (JJ, 1st-year medical student, role-model shadowing reflection).

Despite the immense experience gained from the shadowing activities, not all students had the same success. One student mentioned that she would like to have another opportunity to participate in a shadowing activity, since she did not feel she had an adequate experience with her role model. This result suggests that such activities require preparation from both the students and the teachers serving as role models.

The role models were also given an opportunity to provide comments on the shadowing process and their interactions with the students. All the medical teachers who served as role models in this course provided positive feedback on the process. Close interactions with the students allowed them to discuss the reasoning or background of what they do as practising medical doctors. For example:

*‘Student LL’s positive impressions towards what I do, using her “positive perspectives”, makes me feel honoured. I hope she is able to implement the lessons she learned from this activity. I also hope that she can identify and tell me what I can do better.’* (X, role model, role-model shadowing feedback).

Other role models’ feedback underscored the positive impact of role-model shadowing, as follows:

*‘I think role-model shadowing is a very positive activity since the student can learn what they cannot learn in classrooms; they learn [how to deal] with people and their personalities, and they also learn about [the importance of] integrity in clinical practice.’* (Y, role model, role-model shadowing feedback).

### Field work as experiential learning and a means to implement values in daily practice

All students agreed that their field projects fostered their creativity and encouraged them to experience the challenges of implementing the noble values they learned. The field work also gave students a ‘sense of purpose’, because they were able to help others even with their current capabilities as pre-clinical medical students. They requested more allocated time to conduct their field projects. One student noted:

*‘I learned from this field work that we do not need to wait until we become a great figure to be able to help others. We can start from [having] simple thoughts [and] paying attention to the small details around us.’* (KK, 3rd-year medical student, fieldwork report).

Some students found that they had to be very creative in conducting their projects. They also practised being good and emphatic role models themselves, as they tried to practise the noble values they learned. They also developed their communication skills with people from various backgrounds and age groups, as described in the following quotation:

*‘When I was a tutor at [the] Healthy School [project], I found patience and creativity were very important when teaching about healthy lifestyles. I was expected to make the children understand and able to practise [healthy habits] in their daily lives. As I was not used to dealing with children, it was very exhausting to try to teach them as well as to find fun ways for them to study. I realise now that it is not easy being a tutor.’* (LL, 3rd-year medical student, fieldwork report).

Other students reported that they had to learn how to work effectively in a team to fulfil their project goals, as follows: *‘I realise that teamwork is the key component for a successful project.’* (GG, 1st-year medical student, fieldwork report).

The fieldwork reports were comprised of feedback and comments from the involved parties, which further confirmed that the students participated in valuable activities during this course. Long-term implementation was noted as one of the key challenges.

## Discussion

This phenomenological study aims to explore students’ perceptions of their personal identity formation and professional development following a six-week remediation course to address their lapses in professionalism. The cognitive component of professional development requires an explicit approach and internalisation [10, 11], which were supported by the intensive reflections and small group discussions led by experts. The first theme of this study suggests that the internalisation of noble values and professional attributes requires multiple processes. Students were encouraged to consistently reflect on their learning processes and activities conducted in the course. Students wrote their impressions on the experience and the lessons they learned, thereby prompting them to further consider their strengths and weaknesses in understanding the noble values discussed, such as integrity, altruism, excellence and visionary, as well as the challenges in clinical practice. This metacognitive process facilitated the students’ understanding of the their personal states, as well as their development and identification of future actions to take [13], thereby constructing ‘ways of being’ and ‘ways of acting’ [14]. An analysis of the students’ written reflections was also found useful for identifying gaps in the students’ personal and professional development [36]. The authors argue that, considering the students’ generational characteristics and given their maturity, such activities are more advantageous for personal and professional identity formation when introduced at an early stage of medical training, as well as the remediation process.

In addition, as suggested by the participants, small group activities are thought to be the key factor in supporting medical students’ understanding of the noble values and principles of their personal and professional development. Professionalism is a complex construct to understand [33], certainly for pre-clinical students like those in the present study. The current model of the remediation course enabled intensive interactions (i.e. reciprocal individual and group reflections and feedback) between the students and teachers. In addition to the teacher–student interactions, interactions among the 14 students, who were at different levels of study, seemed to help them discuss the abstract topics from multiple perspectives and understandings. This study confirmed the importance of close interactions between students and teachers as role models in nurturing professional development, starting from the pre-clinical years [18, 23, 36], and a similar approach can serve as the backbone for remediation courses addressing lapses in professionalism. The context of interaction developed in the course also allow for multiple explorations of the students’ understanding of who they are and who they might become [37, 38]. In an Asian context, in which the teacher–student relationship can be very hierarchical [24], the interactions will sometimes need considerable adaptations, thereby allowing both teachers and students to communicate well and appropriately without experiencing any of the awkward feelings of being too distant or too close.

As suggested by Cruess and Cruess [10], medical students, as novices, need support to become full members of the profession. For pre-clinical students, becoming members of the profession is far in the future [17]. Regardless, medical education should nurture the development of complete professional attributes (i.e. ‘doing the right thing’, ‘doing the thing right’ and ‘be the right person doing it’) [3] from an early stage. The second theme in this study highlights the role-modelling process as a crucial component of the personal and professional development of pre-clinical medical students. Role-model shadowing opens the students’ minds to what kind of medical professional they will become in the future (23), be it clinicians, researchers, academics or leaders. As discussed by Cruess et al. [1], there are three types of qualities that should be possessed by a good role model: clinical competence, teaching skills and personal qualities. During the role-modelling process, students witnessed practical applications of the noble values they had been learning about. According to Paice et al. [39] and Passi [12], role modelling is an active process that requires students to observe and evolve. Evolution involves conscious thinking, reflection and abstraction, translating insights into principles and action, and generalisation and behavioural change [12, 39]. Positive role-modelling that allows students to observe, interact and write reflections, as exemplified in the present study, can be a powerful tool for developing students’ personal identity and professional development [16, 40]. Students undergoing remediation may also benefit from the opportunity to develop new insights into their misconduct and translate their revelations into actions for improvement. To ensure the role-modelling process is effective and impactful, both students and teachers should be ready to become actively involved in reflection and discussion. The teachers should also be aware that the behaviours and actions they model, whether positive or negative, will be observed and may be adopted by students [1].

Furthermore, the students were challenged to apply their knowledge and demonstrate their behavioural changes by planning and implementing field projects. These projects enabled them to experience real-world situations, reflect on what they had planned and completed, reconceptualise their understanding of ‘what to become’ and ‘how to become’, and identify future actions. This reflects ‘the process whereby knowledge is created through the transformation of experience. Knowledge results from the combination of grasping and transforming experience’ [41]. The present study also suggests that providing opportunities for real-life experiences accompanied with constructive feedback can be another powerful tool for facilitating personal identity formation and professional development among medical students. What facilitators need to be aware of, however, is whether the students have sufficient knowledge and skills to conduct field projects appropriately [42]. In the remediation course under study, the students were asked to present their field-project proposals prior to implementation so that they could get teacher feedback and so that the teachers could identify each student’s preparedness and each project’s feasibility. Regarding personal identity formation, this approach is critical in facilitating the students’ transition from the pre-clinical classroom into the rich, yet unpredictable learning experiences of field work [13]. Despite receiving very positive feedback from the students upon completing their field projects, the time they were allocated to prepare and finalise their projects was quite limited. The authors found that this is an area for improvement in future implementations, since it is essential for pre-clinical students to conduct long-term projects which allow them to internalise and implement professional values, as well as develop their professional identities, before they become more involved in the community of healthcare practitioners during their clinical rotations.

The authors realised that this study contains other limitations, as it was conducted in only one medical school and involved a small number of participants. The specific characteristics of the participants and the teachers involved in the remediation course may also have influenced the results. The students enrolled in this remediation course were considered to have shown lapses in their responsibilities as medical students. Despite the aim to provide opportunities for them to reflect on their professional development, the authors could not prevent them from feeling they were being punished for their lapses, at least in the beginning of the remediation course. By the end of the course, the students expressed positivity towards their milestone achievements in professional identity development. Considering the impact of the imperial stage on professional identity formation [17], the authors believe that approaches implemented in the remediation course may also benefit other students in pre-clinical studies.

Furthermore, the authors propose that this model has the potential to be applied to medical schools with similar situations, in which students are encouraged to take more initiatives in learning, yet teachers are still regarded to have a significant role in teaching and managing the learning process. The implementation of curricula aimed at professional development is indeed influenced by context [16]. The authors are also aware that implementation of this model requires continuous faculty development, thereby assuring the cognitive–modelling–experiencing cycle benefits the students’ personal identity formation and professional development. The authors fully understand that implementation with a larger cohort requires more than an exponential increase in teachers; in a larger cohort, many other aspects could influence the successful implementation of this model. For example, the authors cannot predict the level of comfort in speaking openly and freely and the consistency of intensive self-reflection and feedback. Also, the relationships between teachers and students in each session and the duration of exposure to the teachers involved may vary.

Given the nature of this remediation course, the authors expect that a small number of students will be enrolled in the future; hence, the overall process can still be conducted well. The authors also recommend future recruitment and training of medical teachers at Universitas Indonesia, should there be a need to implement such an intensive course among a larger cohort of medical students. This way, the power of small group interactions can be maintained. Finally, the authors realise that this remediation course took place over a short period (six weeks); therefore, the long-term impacts of this course still need to be evaluated to assess whether the students are able to internalise and practise the noble values of medicine as future professionals.

Overall, considering the construct of professionalism [33] and students’ characteristics as members of the millennial generation [19–22], the present study offers what the authors call ‘a multimodal approach’ to remediating lapses in professionalism among medical students. This approach incorporates small group discussions and intensive self-reflection processes to internalise and support students’ cognitive understanding of professional and noble values, as well as role-model shadowing and practical experience through field projects. The authors believe that this study may have implications for medical- and health-science educators in two ways. First, this study highlights the cognitive–modelling–experiencing cycle, which can be implemented in the remediation process for lapses in professionalism among medical students. The spirit of remediation includes the realisation that medical students, especially in their pre-clinical years, are still attempting to internalise a professional identity [17]. Second, this study suggests that facilitating the personal and professional identity development of medical students requires intensive interactions between students and teachers, thereby allowing for consistent self-reflections and the provision of feedback which foster socialisation. As such, this approach needs to be maintained and updated in case it is implemented among a larger cohort of medical students in the future.

## Conclusion

Following key theories and evidence in remediating lapses in professionalism among students, the present remediation course facilitates cognitive understanding through small group activities, consistent and intensive self-reflection and feedback to help students internalise values and become self-reflective individuals, and experiential and situated learning through role-model shadowing and field work, which encourages students to experience the realities of being a medical doctor and the challenges faced by those in the healthcare environment. The authors believe that supporting the personal identity formation and professional development of undergraduate medical students is an active, longitudinal and intensive process. Since pre-clinical medical students cannot always relate the professional values they learn to future practice, they may not understand the importance of remediating lapses in professionalism while still enrolled in medical school. Given the results of this study, remediation courses using a multimodal approach has much potential in supporting pre-clinical undergraduate medical students in their personal and professional development. Challenges to implementing the model with a larger cohort have been considered, and studies documenting the long-term impact of this model are required.

## Abbreviation

FMUI: Faculty of Medicine Universitas Indonesia

## References

[1] Cruess SR, Cruess RL (2008). Understanding medical professionalism: A plea for an inclusive and integrated approach. Med Educ.

[2] Epstein RM (1999). Mindful Practice. JAMA.

[3] Harden RM, Crosby JR, Davis MH, Friedman M (1999). AMEE Guide No. 14: Outcome based education: Part 5-From competency to metacompetency: a model for the specification of learning outcomes. Med Teach.

[4] Wilkinson TJ, Wade WB, Knock LD (2009). A blueprint to assess professionalism: Results of a systematic review. Acad Med.

[5] Irby DM, Hamstra SJ (2016). Parting the Clouds: Three Professionalism Frameworks in Medical Education. Acad Med.

[6] Papadakis MA, Hodgson CS, Teherani A, Kohatsu ND (2004). Unprofessional behaviour in medical school is associated with subsequent disciplinary action by a state medical board. Acad Med.

[7] Papadakis MA, Teherani A, Banach MA, Knettler TR, Rattner SL, Stern DT, Veloski JJ, Hodgson CS (2005). Disciplinary action by medical boards and prior behaviour in medical school. NEJM.

[8] Gill AC, Nelson EA, Mian AI, Raphael JL, Rowley DR, Mcguire AL (2015). Responding to moderate breaches in professionalism: An intervention for medical students. Med Teach..

[9] Ziring D, Danoff D, Grosseman S, Langer D, Esposito A, Jan MK, Rosenzweig S, Novack D (2015). How do medical schools identify and remediate professionalism lapses in medical students? A study of U.S. and Canadian medical schools. Acad Med.

[10] Cruess RL, Cruess SR (2006). 2006. Teaching professionalism: general principles. Med Teach.

[11] Maudsley G, Strivens J (2004). 2004. Promoting professional knowledge, experiential learning & critical thinking for medical students. Med Educ.

[12] Passi V, Johnson S, Peile E, Wright S, Hafferty F, Johnson N (2013). Doctor role modelling in medical education: BEME Guide No. 27. Med Teach.

[13] Sandars J (2009). The use of reflection in medical education: AMEE Guide No. 44. Med Teach.

[14] Sharpless J, Baldwin N, Cook R, Kofman A (2015). The becoming: Students’ reflections on the process of professional identity formation in medical education. Acad Med.

[15] Wilson I, Cowin LS, Johnson M, Young H (2013). Professional identity in medical students: pedagogical challenges to medical education. Teach Learn in Med.

[16] Guraya SS, Almaramhy HH (2016). The legacy of teaching medical professionalism for promoting professional practice. Biomed Pharmacol J.

[17] Cruess RL, Cruess SR, Boudreau D, Snell L, Steinert Y (2015). A Schematic representation of the professional identity formation and socialization of medical students and residents: A guide for medical educators. Acad Med.

[18] Baernstein A, AEA O, Chang TA, Wenrich MD (2009). Learning professionalism: Perspectives of preclinical medical students. Acad Med.

[19] Borges NJ, Manuel S, Elam CL, Jones BJ (2006). Comparing millennial and generation X medical students at one medical school. Acad Med.

[20] Blue AV, Crandall S, Nowacek G, Luecht R, Chauvin S, Swick H (2009). Assessment of matriculating medical students’ knowledge and attitudes towards professionalism. Med Teach.

[21] Borges NC, Manuel SR, Elam CL, Jones BJ (2010). Differences in motives between millenial and generation X medical students. Med Educ.

[22] DiLullo C, McGee P, Kriebel RM (2011). Demystifying the millennial student: A reassessment in measures of character and engagement in professional education. Anat Sci Educ.

[23] Guraya SY, Norman RI, Roff S (2016). Exploring the climates of undergraduate professionalism in a Saudi and a UK medical school. Med Teach.

[24] Ho MJ, Yu KH, Hirsh D, Huang TS, Yang PC (2011). Does one size fit all? Building a framework for medical professionalism. Acad Med.

[25] Chandratilake M, McAleer S, Gibson J (2012). Cultural similarities and differences in medical professionalism: A multi-region study. Med Educ.

[26] Jha V, McLean M, Gibbs TJ, Sandars J (2015). 2015. Medical professionalism across cultures: A challenge for medicine and medical education. Med Teach.

[27] Hodges BD, Ginsburg S, Cruess R, Cruess S, Delport R, Hafferty F, Ho MJ, Holmboe E, Holtman M, Ohbu S (2011). Assessment of professionalism: Recommendations from the Ottawa 2010 Conference. Med Teach.

[28] Tsugawa Y, Tokuda Y, Ohbu S (2009). Professionalism Mini-Evaluation Exercise for medical residents in Japan: A pilot study. Med Educ.

[29] Chiu CH, Arrigo LG, Tsai D (2009). Historical context for the growth of medical professionalism and curriculum reform in Taiwan. Kaohsiung J Med Sci.

[30] Haque M, Zulkifli Z, Haque SZ, Kamal ZM, Salam A, Bhagat V, Alattraqchi AG, Rahman NIA (2016). Professionalism perspectives among medical students of a novel medical graduate school in Malaysia. Adv in Med Educ Prac.

[31] Hauer KE, Ciccone A, Henzel TR (2009). Remediation of the deficiencies of physicians across the continuum from medical school to practice: A thematic review of the literature. Acad Med.

[32] Indonesian Medical Council (2012). Standard of competence for Indonesia Medical Graduates.

[33] Hafferty FW, Castellani B (2010). The increasing complexities of professionalism. Acad Med.

[34] Creswell JW (2013). Qualitative inquiry and research design: Choosing among five approaches.

[35] Starks H, Trinidad SB (2007). Choose your method: A comparison of phenomenology, discourse analysis and grounded theory. Qual Health Res.

[36] Howe A, Barret A, Leinster S (2009). How medical students demonstrate their professionalism when reflecting on experience. Med Educ.

[37] Baernstein A, Fryer-Edwards K (2003). Promoting reflection on professionalism: A comparison trial of educational interventions for medical students. Acad Med.

[38] Monroux LV (2010). Identity, identification and medical education: Why should we care?. Med Educ.

[39] Paice E, Heard S, Moss F (2002). How important are role models in making good doctors?. BMJ.

[40] Passi V, Johnson N (2016). The impact of positive doctor role modeling. Med Teach.

[41] Kolb DA (1984). Experiential learning: Experience as the source of learning and development.

[42] Vågan A (2009). Medical students’ perceptions of identity in communication skills training: a qualitative study. Med Educ.

